# High Hepcidin expression predicts poor prognosis in patients with clear cell renal cell carcinoma

**DOI:** 10.1186/s13000-022-01274-9

**Published:** 2022-12-31

**Authors:** Yuting Tang, Shengdong Ge, Xiao Zheng, Jiejiao Zheng

**Affiliations:** 1 Department of Rehabilitation, Municipal Hospital of Traditional Chinese Medicine, Affiliated to Shanghai University of Traditional Chinese Medicine, Shanghai, 200071 People’s Republic of China; 2 Department of Urology, The First School of Clinical Medicine, Nanfang Hospital, Southern Medical University, Southern Medical University, Guangzhou, China; 3 Department of Rehabilitation, HuaDong Hospital, FuDan University, Shanghai, 200040 People’s Republic of China

**Keywords:** Clear cell renal cell carcinoma, HAMP, Bioinformatics analysis, Prognosis, Immune cell infiltration

## Abstract

Clear cell renal cell carcinoma (ccRCC) is a growing public health challenge worldwide. Hepcidin antimicrobial peptide (HAMP) is differentially expressed in various tumors. However, the roles and functions of HAMP in ccRCC remain unclear. In the present study, we integrated systematic bioinformatics approaches to investigate the roles and functions of HAMP and its association with immune cell infiltration in ccRCC. Compared with paracancerous tissue, HAMP expression was significantly upregulated in ccRCC patients. Meanwhile, we found good diagnostic performance of HAMP for ccRCC patients and its close associations with the clinicopathological features of ccRCC patients. In addition, we found that HAMP is closely related to multiple immune pathways and positively correlated with various immune cells. HAMP was a significant independent predictor for ccRCC. High expression of HAMP was associated with worse clinical prognosis and more immune cell infiltration in ccRCC patients. HAMP may offer potential as a biomarker to predict prognosis and the clinical treatment outcome of ccRCC patients.

**Supplementary Information:**

The online version contains supplementary material available at 10.1186/s13000-022-01274-9.

## Introduction

According to the latest cancer statistics, renal cell carcinoma (RCC) remains one of the leading causes of cancer-related death in urological tumors; approximately 76,080 new cases of RCC and 13,780 patients die from the disease in America in 2021 [1]. As we all know, there are various subtypes of RCC. The most common clinical pathological type of RCC is clear cell renal cell carcinoma (ccRCC), which is approximately 85%, followed by papillary renal cell carcinoma (pRCC), chromophobe renal cell carcinoma (chRCC) and others[2, 3]. With advances in medicine, the overall survival of ccRCC patients has improved [4]. However, ccRCC is insensitive to chemotherapy and radiotherapy in the clinic [5]. Approximately one-third of ccRCC patients experience recurrence and metastasis after surgical resection, which is the main cause of death. The mean survival period of these patients is usually less than one year [6]. The tumorigenic mechanism of ccRCC is truly complicated. Although several studies have shown that many genetic changes and epigenetic dysregulations are associated with the development and progression of ccRCC, the molecular mechanism of renal cell carcinoma pathogenesis is still unclear and needs to be further studied [7, 8]. To date, no specific and sensitive molecular biomarkers for ccRCC have been declared. Therefore, continued efforts to identify novel biomarkers for predicting tumor progression and guiding the most suitable treatment for ccRCC patients are urgently needed.

Hepcidin antimicrobial peptide (HAMP), a preprotein of 84 amino acids composed of a signal peptide, regulates iron homeostasis via ferroportin inactivation and exhibits bactericidal and fungicidal properties in vitro [9, 10]. The major role of HAMP is the regulation of iron metabolism by inhibiting the posttranslational iron exporter ferroportin [11, 12] (Figure S1). HAMP is mainly produced in the liver, but recent studies have shown that HAMP is also produced in other tissues: kidney [13], macrophages [14], and pancreatic beta cells [15]. HAMP synthesis is significantly induced by iron overload and infection or inflammation and inhibited by iron deficiency and hypoxia [16]. A considerable proportion of studies have shown that iron homeostasis is one of the markers of tumor cell metabolism, playing an important role in tumors occurrence and development [17, 18]. Interestingly, previous studies reported that HAMP was downregulated in cholangiocarcinoma [19] and liver hepatocellular carcinoma [20] and upregulated in breast cancer, pancreatic adenocarcinoma [21], prostate cancer, and colorectal adenocarcinoma [22] However, the roles of HAMP and its association with immune cell infiltration in ccRCC are unexplored.

In the present study, we aimed to integrate systematic bioinformatics approaches to investigate the roles of HAMP and its association with immune cell infiltration in ccRCC. Compared with paracancerous tissue, HAMP expression was significantly upregulated in ccRCC patients. Moreover, we validated the diagnostic performance of HAMP for ccRCC patients and its close associations with the clinicopathological features of ccRCC patients. In addition, we explored the relationship between HAMP and immune cell infiltration, and preliminarily explored whether HAMP has a certain role in immunotherapy. Our study observations emphasized a prominent role of HAMP in ccRCC and uncovered that HAMP represented a significant independent predictor for ccRCC. Taken together, HAMP is promising as a novel biomarkers for predicting tumor progression and guiding the suitable treatment for ccRCC patients.

## Materials and methods

### Data collection and analysis

We explored the pivotal roles and underlying functions of HAMP in ccRCC using comprehensive bioinformatics analysis methods. First, we downloaded the RNA-sequencing data and the corresponding clinical data of TCGA pancancer data and GTEx from the UCSC Xena database (https://xenabrowser.net/datapages/). The expression data were log2[TPM (transcripts per million) + 1] transformed for normalization by the “RNA-Seq by ExpectationMaximization” package. We executed differential expression analysis of HAMP between tumor and adjacent normal samples for the different tumors or specific tumor subtypes in The Cancer Genome Atlas (TCGA) and GTEx databases. Next, we analyzed the expression of HAMP in RCC tumors, including ccRCC, pRCC, and chRCC. The diagnostic efficiency of HAMP in ccRCC, pRCC, and chRCC were excavated. Then, we obtained three normalized independent microarray datasets, namely, GSE53757, GSE66272, and GSE105261, from the Gene Expression Omnibus (GEO; https://www.ncbi.nlm.nih.gov/geo/) database. Additionally, we obtained box plots of the HAMP expression level in different clinicopathologic features, including age, pathological stages, pathological T stages, pathological N stages, and pathological M stages. In the present study, all data were acquired from public Online databases. Hence, ethical approval and informed consent of the patients were not required.

### Human tissue samples

We used the ccRCC samples from the The First Affiliated Hospital of Anhui Medical University (Hefei, China), between 2021 and 2022, that our group had previously collected. The present study conformed to the standards of the Declaration of Helsinki and was approved by the Ethics Committee of Human Research of The First Affiliated Hospital of Anhui Medical University (No. PJ2019-14–22).

### RNA Extraction and qRT–PCR

Total RNA was extracted with TRIzol Reagent (Invitrogen, USA). Quantification of HAMP and GAPDH was performed with the SYBR® PrimeScript™ RT-PCR Kit (Takara, Japan). The primer sequences for HAMP and GAPDH were used: HAMP primers forward: 5’-CACAACAGACGGGACAACTT-3’, reverse: 5’-CGCAGCAGAAAATGCAGATG-3’; GAPDH primers forward: 5’-GGGAGCCAAAAGGGTCAT-3’, reverse: 5’-GAGTCCTTCCACGATACCAA-3’. Gene expression was normalized to that of GAPDH.

### Western Blotting

Cells and tissues were homogenized and lysed with ice-cold RIPA buffer supplemented with a proteasome and phosphatase inhibitor (#P0013B, Beyotime, China) for 30 min, and total proteins were extracted. The procedure for standard western blotting was performed as described in a previous study [23]. Primary antibodies anti-GAPDH (1:1000, #2118; CST) and anti-HAMP (1:1000, BM5068; Boster) were used to determine the expression of the corresponding proteins. Membranes were incubated in the corresponding horseradish peroxidase (HRP)-conjugated secondary antibody at a 1:3000 ratio (Affinity Biosciences, Cincinnati, USA), and the protein bands were visualized by an ECL western blotting detection kit (Beyotime, Shanghai, China). GAPDH was used as the internal reference to normalize the protein loading. The images were quantified using ImageJ software.

### Survival analysis

We used the “Survival” R package (https://cran.r-project.org/web/packages/survival/index.html) to obtain the OS (overall survival), DSS (disease-specific survival), and PFI (progression-free interval) survival map data of HAMP in RCC tumors. The cutoff-high (50%) and cutoff-low (50%) values of HAMP expression were used as thresholds in TCGA database. To further determine the effect of HAMP expression in ccRCC patients, we use univariate Cox regression analysis for calculating the association between HAMP expression and patient’s OS in TCGA database. Then, a multivariate Cox regression analysis was executed to assess if the HAMP is an independent prognostic factor for ccRCC patient survival. A *P* value < 0.05 was considered a significant difference.

### Protein–protein interaction (PPI) network comprehensive analysis

Importing the HAMP into the online tool STRING (https://string-db.org/), which hosts a big collection of integrated and consolidated protein–protein interaction data, we obtained the PPI network information. The confidence score > 0.7 was considered significant.

### HAMP-related gene functional enrichment analysis

To explore HAMP-related biological pathways, genes that were strongly correlated with HAMP expression were obtained (correlation coefficient R > 0.4). We used the “DESeq2” R package (http://www.bioconductor.org/packages/release/bioc/html/DESeq2.html) to identify the differentially expressed genes for the high and low HAMP expression groups in the TCGA database. A |log fold change (FC)|> 1 and adjusted P value < 0.05 were set as threshold values. Subsequently, we performed gene ontology (GO) and Kyoto Encyclopedia of Genes and Genomes (KEGG) enrichment analyses to investigate the underlying molecular mechanisms of the HAMP gene using the “Clusterprofiler” R package.

### Gene set enrichment analysis (GSEA)

To further explore the potential molecular mechanisms affected by HAMP in ccRCC, we obtained the potential signaling pathway associated with ccRCC between the high and low HAMP expression groups using the “clusterProfiler” R package (http://www.bioconductor.org/packages/release/bioc/html/) for GSEA. We selected the “h.all.v7.1.symbols.gmt” file as the reference gene set file with the threshold values as adjusted *P* value < 0.05 and false discovery rate (FDR) < 0.25.

### Immune infiltration analysis

Immune infiltration analysis of ccRCC patients was employed by single-sample gene set enrichment analysis (ssGSEA) using the “GSVA” R package (http://www.biocondutor.org/package/release/bioc/html/GSVA.html) to quantify the 24 types of immune cells based on the metagenes, comparing activated DCs (aDCs), B cells, CD8 + T cells, cytotoxic cells, dendritic cells (DCs), eosinophils, immature DCs (iDCs), macrophages, mast cells, neutrophils, NK CD56bright cells, NK CD56dim cells, natural killer (NK) cells, plasmacytoid DCs (pDCs), T cells, Th cells, T effector memory cells (Tem), T follicular helper cells (Tfh), T gamma delta cells (Tgd), Th1 cells, Th2 cells, Th17 cells, T regulatory cells (Treg).

### Gene correlation analysis

In the module “Correlation Analysis” of The Gene Expression Profiling Interactive Analysis (GEPIA) (http://gepia.cancer-pku.cn/index.html), which is a useful web portal for gene expression analysis based on TCGA and GTEx data, we input HAMP and immune markers into the “gene” frame. To intensely explore the possible role of HAMP in the infiltration of various immune cells in ccRCC, we explored the relationships between HAMP expression with multiple markers for immune cells with the option of Spearman’s method and matching TCGA and GTEx data and log2 (TPM) for log-scale. In addition, we used The Tumor Immune Estimation Resource (TIMER), a public website which covers 32 cancer types and encompasses 10,897 samples from TCGA database, to validate the genes which were of significant correlation with HAMP expression in the GEPIA.

### Statistical analysis

We performed the Wilcoxon rank-sum test and Wilcoxon rank signed test to analyze the expression of HAMP in nonpaired samples and paired samples, respectively. Univariate and multivariate Cox regression analyses were used to evaluate the prognostic values of the clinicopathologic features and HAMP expression. Furthermore, we used Kaplan–Meier curves and log-rank tests to identify the survival difference of ccRCC patients. All procedures were conducted using R software (Version 3.6.3). In all tests, the results were considered to be significant if the *P* value was < 0.05.

## Results

### HAMP expression analysis

**Fig. 1:**
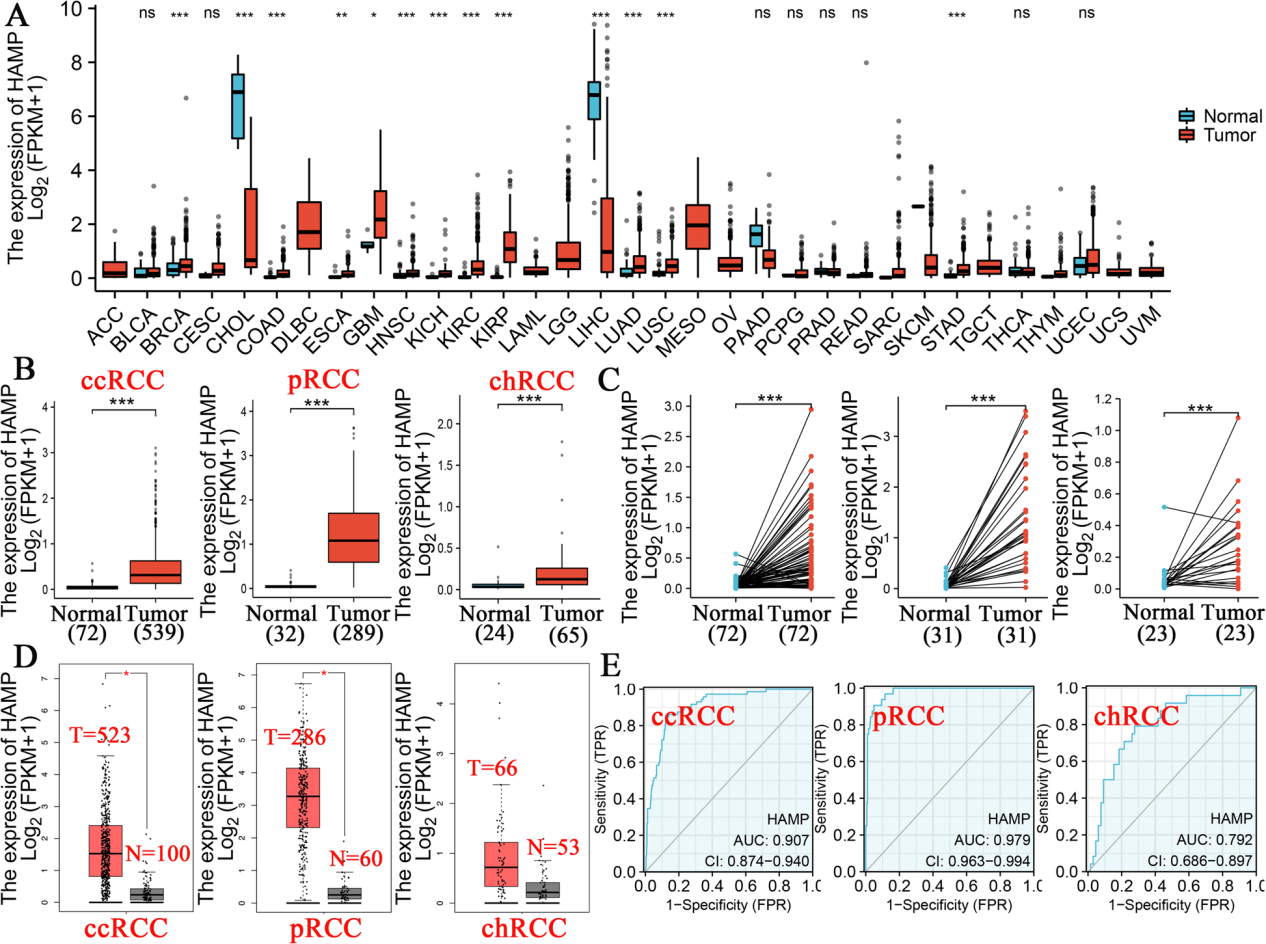
Expression of hepcidin in renal cell carcinoma. **A** HAMP expression in different types of cancer was investigated in the TCGA database. **B** Analysis of HAMP expression in renal cell carcinoma tissues (ccRCC, pRCC, and chRCC) and adjacent normal tissues in the TCGA database. **C** TCGA database and statistical analyses of HAMP expression in pairs of renal cell carcinoma (ccRCC, pRCC, and chRCC) and adjacent normal tissues. **D** Analysis of HAMP expression in renal cell carcinoma (ccRCC, pRCC, and chRCC) and adjacent normal tissues in the TCGA and GTEx databases. **E** ROC analysis of HAMP shows promising discrimination power between tumor and normal tissues for renal cell carcinoma (KIRC, KIRP, and KICH). ns: *P* > 0.05, *P < 0.05, **P < 0.01, ***P < 0.001, ****P < 0.0001

We first assessed HAMP expression in pancancer data from TCGA. The results found that the expression level of HAMP was significantly higher in eleven tumors, including breast cancer, colon adenocarcinoma, esophageal carcinoma, glioblastoma multiforme, head and neck squamous cell carcinoma, ccRCC, pRCC, and chRCC, lung adenocarcinoma, lung squamous cell carcinoma, and stomach adenocarcinoma. In contrast, the expression level of HAMP was significantly lower in cholangiocarcinoma and liver hepatocellular carcinoma (Fig. 1A). Next, we found that the expression level of HAMP in RCC tissue was substantially and significantly higher than that in para-carcinoma tissues (Fig. 1B). Similarly, HAMP expression levels were also higher in tumor tissues than in paired para-carcinoma tissues (Fig. 1C). In addition, we implemented performed HAMP expression analysis of RCC in TCGA and GTEx databases. The results showed that HAMP expression levels were higher in ccRCC and pRCC (*P* < 0.05; Fig. 1D), yet there was no significant difference in chRCC (*P* > 0.05; Fig. 1D). Meanwhile, HAMP expression showed promising discriminative power in RCC (ccRCC, pRCC, and chRCC) with area under the curve values of 0.907, 0.979, and 0.792, respectively (Fig. 1E).

### Validation of HAMP expression levels in tumor and paracancer tissue by Western Blotting and qRT-PCR analysis

**Fig. 2:**
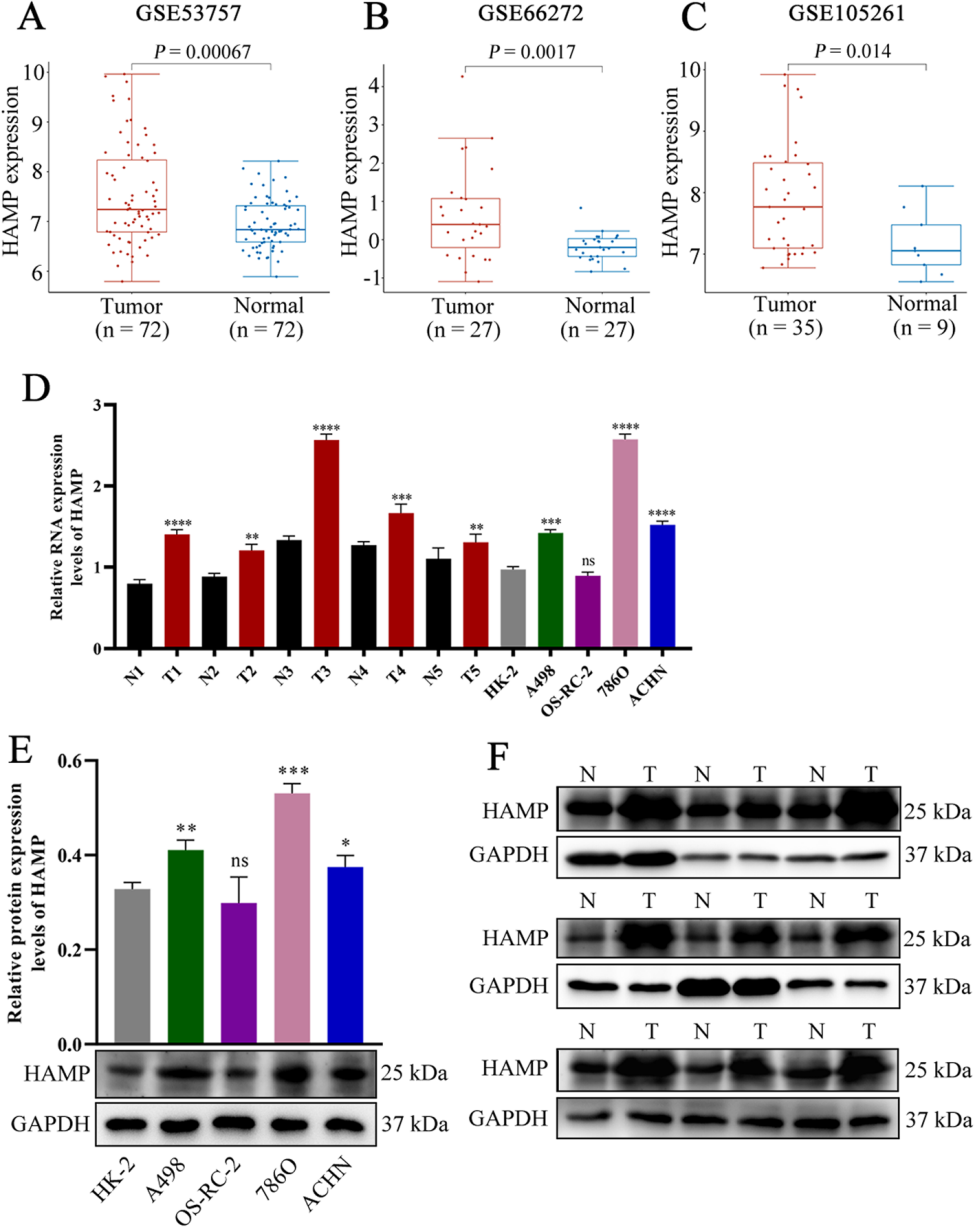
Validation the expression of hepcidin in clear cell renal cell carcinoma. In GSE53757 (**A**), GSE66272 (**B**), and GSE105261 (**C**) datasets, the expression levels of HAMP in renal cell carcinoma paracarcinoma tissue and normal tissues. **D** The mRNA expression levels of HAMP in clear cell renal cell carcinoma tissues and cell lines. **E** Protein expression levels of HAMP in clear cell renal cell carcinoma cell lines. **F** Protein expression levels of HAMP in patients with clear cell renal cell carcinoma. ns: *P* > 0.05, *P < 0.05, **P < 0.01, ***P < 0.001, ****P < 0.0001

Three normalized independent microarray datasets, namely, GSE53757 (Figure S2), GSE66272 (Figure S3), and GSE105261 (Figure S4). We explored the expression levels of HAMP in ccRCC paracarcinoma tissue and normal tissues in GSE53757, GSE66272, and GSE105261 datasets (Fig. 2A, 2B, 2C). This is consistent with the results of previous analyses, the results showed that the expression level of HAMP in RCC tissue was substantially and significantly higher than that in para-carcinoma tissues. In addtion, we found the mRNA expression levels of HAMP in ccRCC’ tissues and cell lines was higher than normal tissue and HK-2 cell by qRT-PCR analysis, respectively (Fig. 2D). Meanwhile, the Western Blotting analysis showed that protein expression levels of HAMP in ccRCC’ tissues and cell lines was higher than normal tissue and HK-2 cell analysis, respectively (Fig. 2E, 2F).

### Associations between HAMP expression and clinicopathologic features

**Fig. 3:**
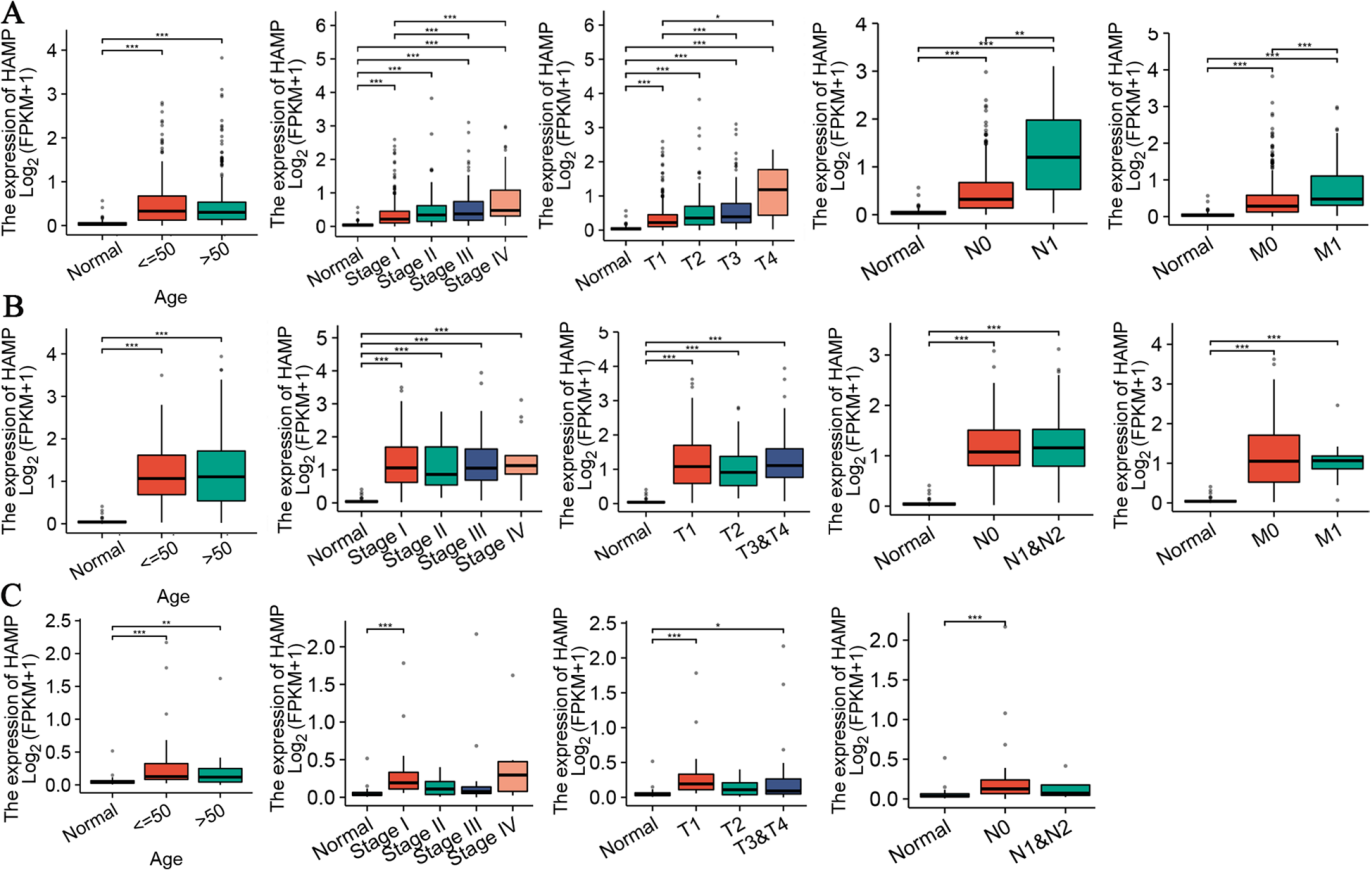
Box plots showed the association of HAMP expression with clinicopathologic characteristics in renal cell carcinoma, including age, pathological stage, pathological T stage, pathological N stage, and pathological M stage. **A** Kidney renal clear cell carcinoma (KIRC); **B** Kidney renal papillary cell carcinoma (KIRP); **C** Kidney chromophobe (KICH). ns: *P* > 0.05, *P < 0.05, **P < 0.01, ***P < 0.001, ****P < 0.0001

We evaluated the association between the expression levels of HAMP in different clinical subgroups in RCC. The bar charts showed that KIRC patients tended to have a higher age, higher pathological stage, higher pathological T stage, higher pathological N stage, and more node metastasis in different clinical subgroups (Fig. 3A). Although the expression levels of HAMP in pRCC and chRCC were higher in the tumor subgroup than in normal tissues, there were no significant differences between the different clinical subgroups (Fig. 3B, 3C). The association between HAMP expression and the clinicopathologic features of ccRCC is shown in Table 1.

**Table 1:** Association between HAMP expression and clinicopathologic features of ccRCC

Characteristic	Low expression of HAMP	High expression of HAMP	*p*
n	265	265	
Age, n (%)			0.339
< = 60	126 (23.8%)	138 (26%)	
> 60	139 (26.2%)	127 (24%)	
Gender, n (%)			0.003
Female	110 (20.8%)	76 (14.3%)	
Male	155 (29.2%)	189 (35.7%)	
Pathologic stage, n (%)			< 0.001
Stage I	165 (31.3%)	100 (19%)	
Stage II	24 (4.6%)	33 (6.3%)	
Stage III	53 (10.1%)	70 (13.3%)	
Stage IV	22 (4.2%)	60 (11.4%)	
T stage, n (%)			< 0.001
T1	166 (31.3%)	105 (19.8%)	
T2	27 (5.1%)	42 (7.9%)	
T3	70 (13.2%)	109 (20.6%)	
T4	2 (0.4%)	9 (1.7%)	
N stage, n (%)			0.009
N0	118 (46.3%)	121 (47.5%)	
N1	2 (0.8%)	14 (5.5%)	
M stage, n (%)			< 0.001
M0	224 (45%)	196 (39.4%)	
M1	21 (4.2%)	57 (11.4%)	
OS event, n (%)			< 0.001
Alive	207 (39.1%)	150 (28.3%)	
Dead	58 (10.9%)	115 (21.7%)	
DSS event, n (%)			< 0.001
Alive	227 (43.7%)	184 (35.5%)	
Dead	33 (6.4%)	75 (14.5%)	
PFI event, n (%)			0.002
Alive	202 (38.1%)	168 (31.7%)	
Dead	63 (11.9%)	97 (18.3%)	

### Identification of HAMP with prognostic significance in ccRCC

**Fig. 4:**
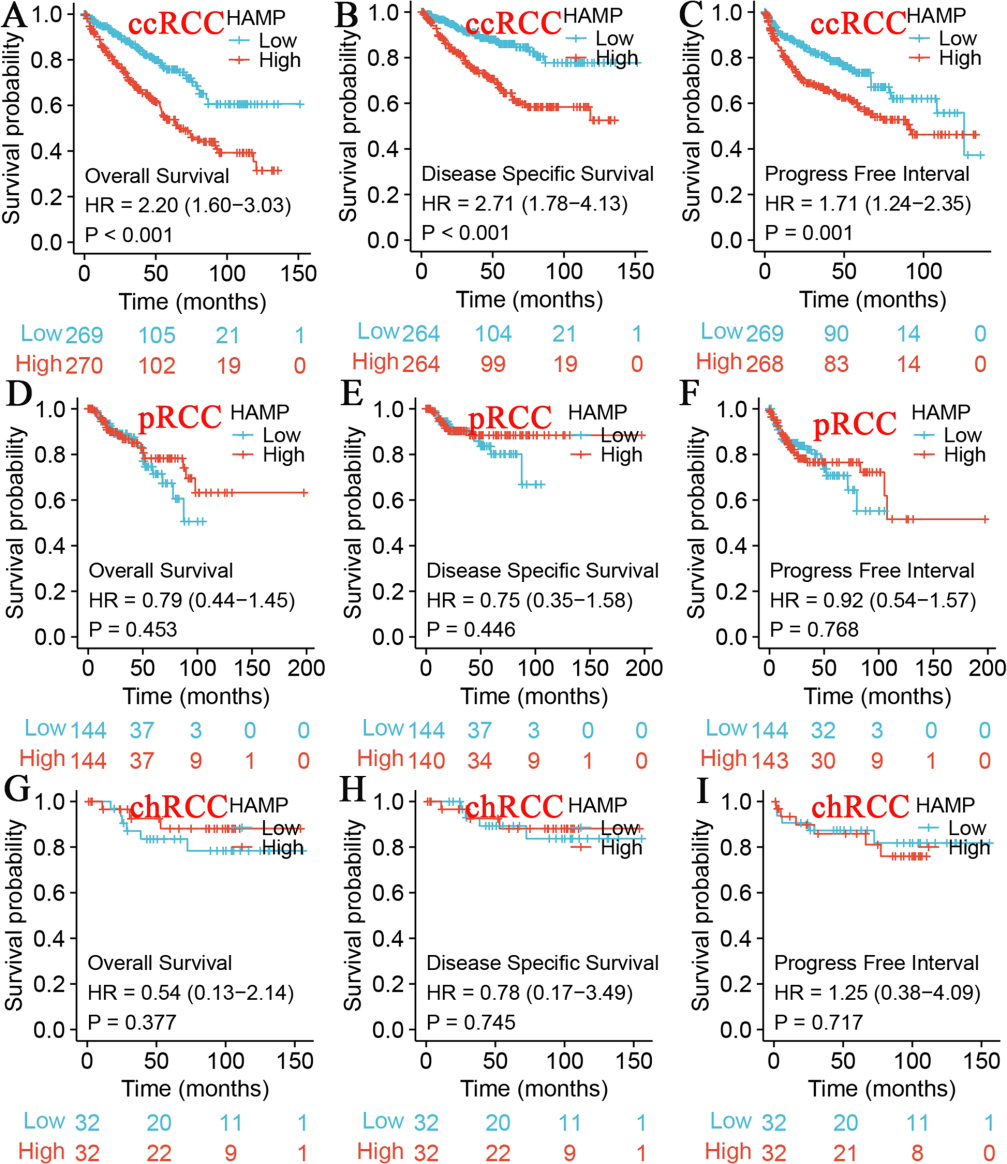
Kaplan–Meier survival curves comparing the high and low expression of HAMP in renal cell carcinoma, including overall survival, disease-specific survival, and progression-free interval. **A** Kidney renal clear cell carcinoma (ccRCC); **B** Papillary renal cell carcinoma (pRCC); **C** Chromophobe renal cell carcinoma (chRCC)

Kaplan–Meier curves showed that high HAMP expression was more strongly associated with worse OS, DSS, and PFI in ccRCC (*P* < 0.001; *P* < 0.001; *P* = 0.001; Fig. 4A). In contrast, there were no significant differences in pRCC and chRCC (Fig. 4B, 4C). These findings demonstrated that HAMP expression is related to the prognosis of ccRCC and revealed that HAMP may play an important regulatory role in ccRCC progression. The ROC curve showed that HAMP expression had good performance for survival prediction of ccRCC patients in TCGA dataset, and the area under the curve (AUC) values for the 1-year, 3-year and 5-year overall survivals were 0.681, 0.633, and 0.626, respectively(Figure S5).

**Fig. 5:**
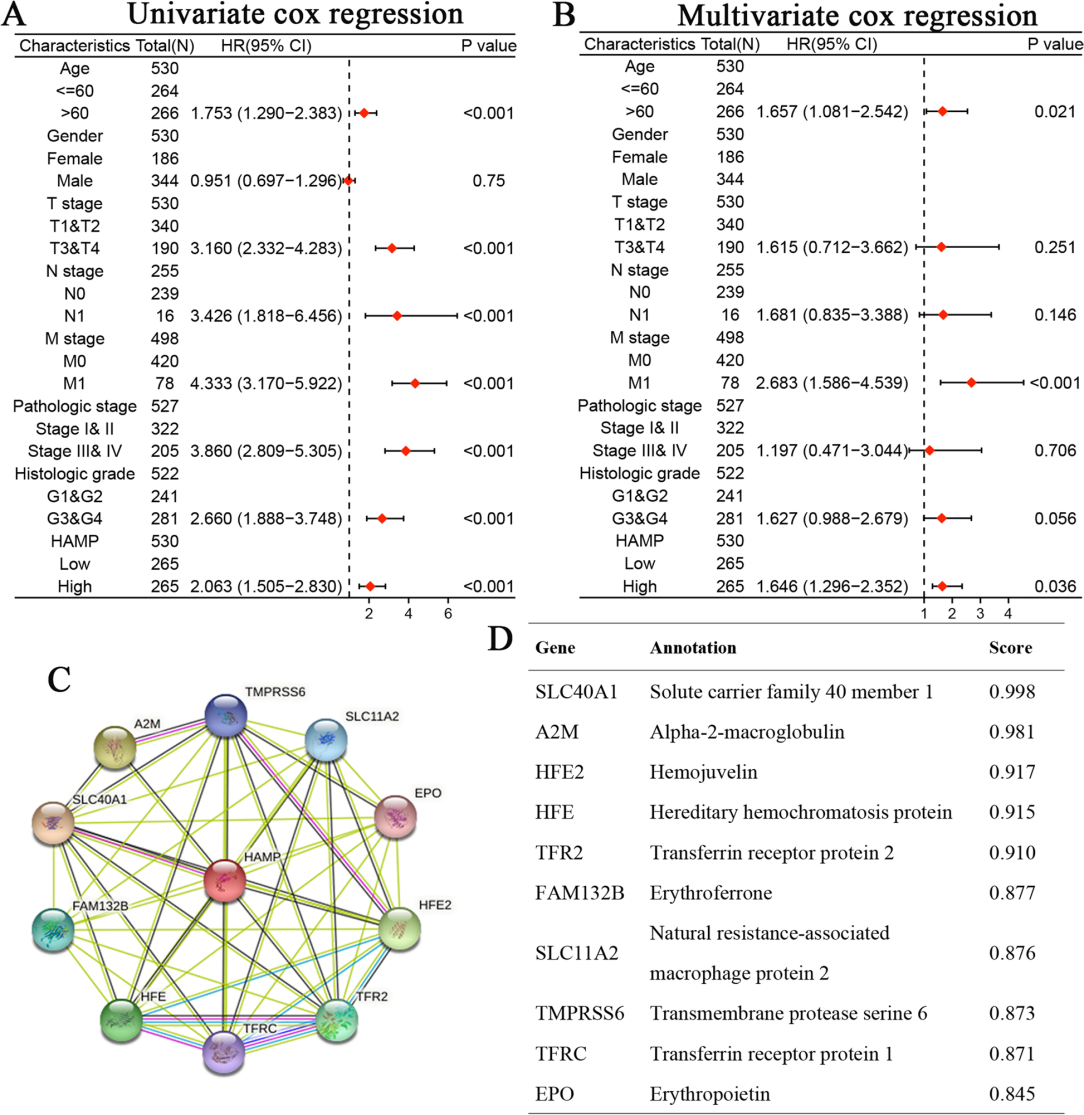
Univariate and multivariate regression analysis of HAMP and other clinicopathologic parameters with OS in ccRCC patients (**A**, **B**). HAMP-interaction proteins in ccRCC tissue (**C**). Annotation of HAMP-interacting proteins and their co-expression scores (**D**)

Based on the value of HAMP in ccRCC, we explored the relationship between HAMP expression and the clinical characteristics of patients with ccRCC using univariate Cox regression analysis. The results showed that age, pathologic T stage, pathologic N stage, pathologic M stage, pathologic stage, histologic grade, and HAMP expression were correlated with poor OS. Subsequently, we demonstrated that HAMP expression was an independent prognostic predictor using multivariate Cox regression analysis (Fig. 5A, B).

### Constructing protein interaction networks

Functional interaction between proteins are essential for the molecular mechanism and metabolism of malignant tumors. We used STRING online tool to analyze the PPI network of HAMP protein to determine their interactions in the progression of ccRCC. The top 10 proteins and corresponding gene names, annotations and scores are listed in Fig. 5C, D.

### Identification of DEGs between the high and low HAMP expression groups

We performed differential expression analysis between the groups with high and low HAMP expression using the “DSEeq2” R package. In all, 234 upregulated and 139 downregulated genes were identified in the HAMP high expression group (Figure S6A). The heatmap of the 20 genes with the highest correlation with HAMP expression is shown in Figure S6B. Meanwhile, GEPIA analysis demonstrated that GPR84, FCGR1A, RNASE2, FCGR1B, STAC3, FCGR1G, ARPC1B, OSCAR, SPI1, and LILRB4 were increased in ccRCC (*P* < 0.05; Figure S7). Additionally, these genes were significantly related to the OS of ccRCC patients, except FCGR1G and LILRB4 (Figure S8).

### Functional enrichment analysis of HAMP-related partners

**Fig. 6:**
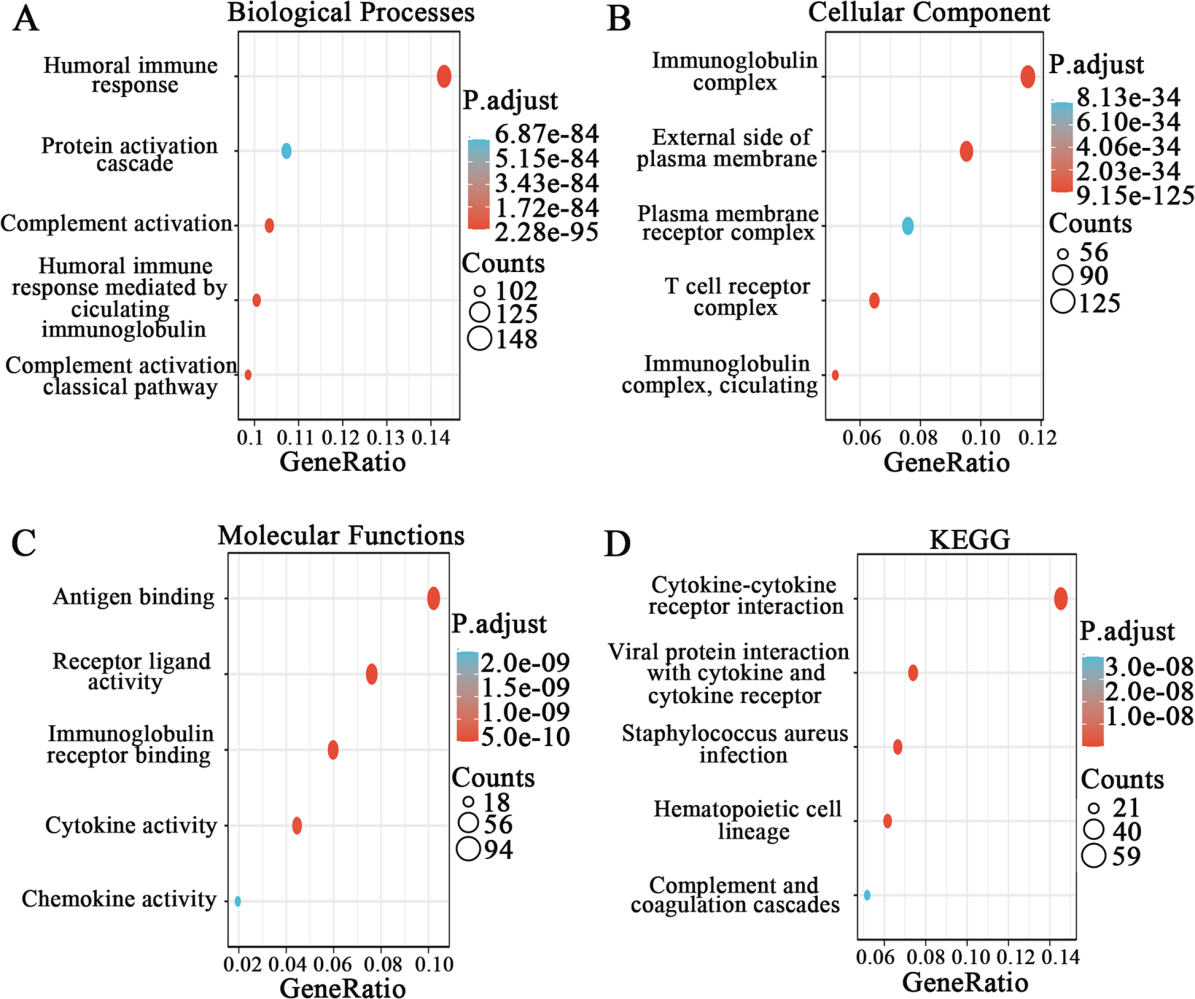
HAMP-related functional enrichment analysis. **A** Enriched GO terms in the “biological process” category. **B** Enriched GO terms in the “cellular component” category. **C** Enriched GO terms in the “molecular function” category. **D** KEGG analysis of the identified potential functions and pathways. The x-axis represents the proportion of differentially expressed genes (DEGs), and the y-axis represents enrichment pathways. Different circle sizes represent the number of DEGs

To further understand the potential role of HAMP in ccRCC, GO and KEGG analyses were executed on HAMP coexpressed genes (Supplementary Table 3). The top five results of the GO analyses are shown in Fig. 6A-C. The results showed that the significantly enriched terms were “humoral immune response”, “protein activation cascade”, “complement activation”, “humoral immune response mediated by circulating immunoglobulin”, and “complement activation classical pathway” for biological processes, “immunoglobulin complex”, “external side of plasma membrane”, “plasma membrane receptor complex”, “T cell receptor complex”, “immunoglobulin complex circulating” for cellular component, “antigen binding”, “receptor ligand activity”, “immunoglobulin receptor binding”, and “cytokine activity”, “chemokine activity” for molecular functions. The top five significantly enriched terms in the KEGG pathway were “cytokine–cytokine receptor interaction”, “viral protein interaction with cytokine and cytokine receptor”, “Staphylococcus aureus infection”, “hematopoietic cell lineage”, and “complement coagulation cascades” (Fig. 6D).

### HAMP-related signaling pathways based on GSEA

**Fig. 7:**
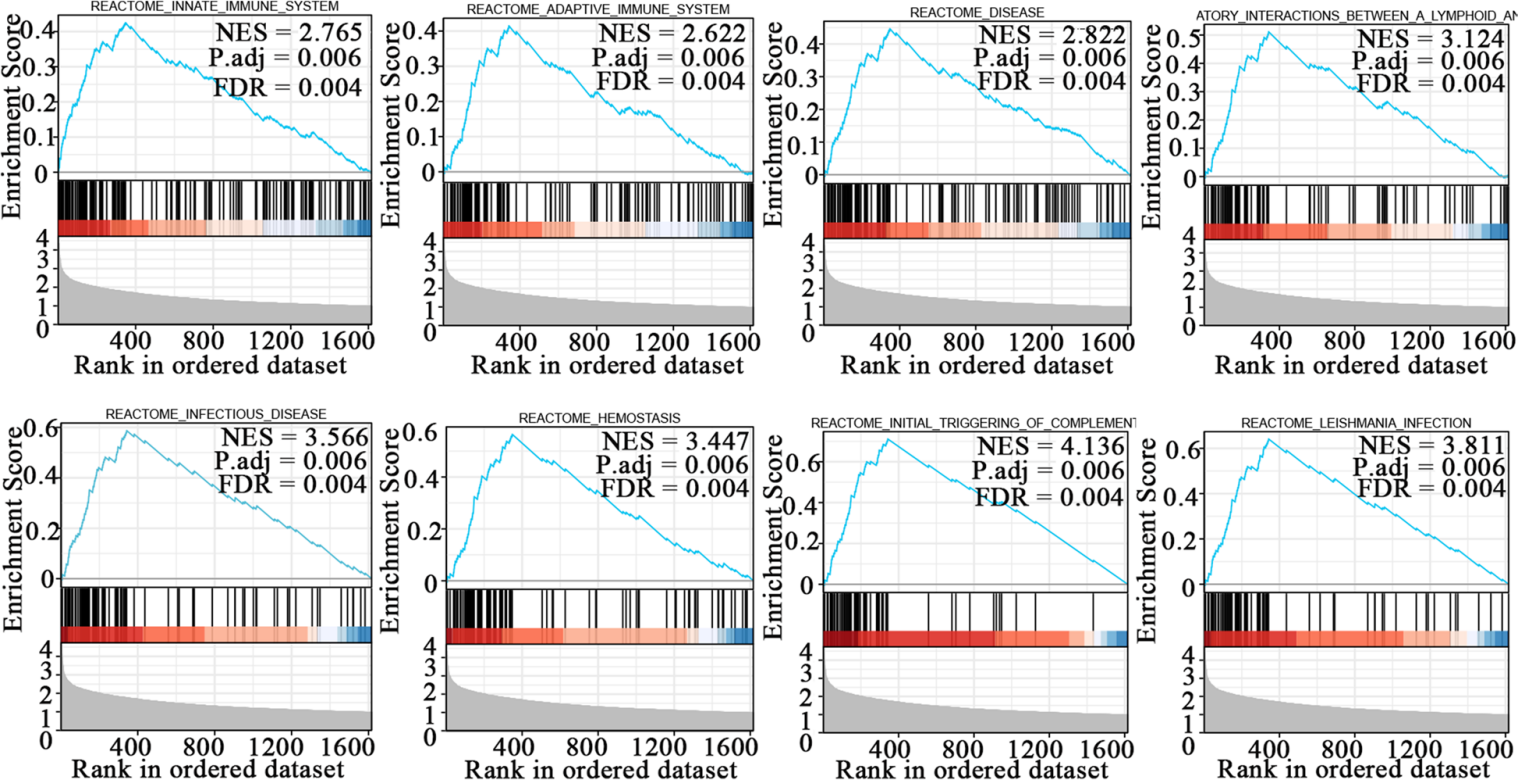
Enrichment plots from GSEA. Several pathways were differentially enriched in ccRCC patients according to high and low HAMP expression. **A** Innate immune system; **B** adaptive immune system; **C** disease; **D** immunoregulatory interactions between a lymphoid and a nonlymphoid cell; **E** infectious disease; **F** hemostasis; **G** initial triggering of complement; **H** Leishmania infection. NES, normalized enrichment score; ADJ p-Val, adjusted P value; FDR, false discovery rate

To further explore the potential molecular mechanisms affected by HAMP in ccRCC, we obtained the potential signaling pathway associated with ccRCC between the high and low HAMP expression groups using the “clusterProfiler” R package for GSEA (adjusted *P* value < 0.05, |log FC|> 1; Supplementary Table 4). The top eight significantly different pathways were innate immune system (normalized enrichment score (NES) = 2.765, size = 149), adaptive immune system (NES = 2.622, size = 121), disease (NES = 2.822, size = 120), immunoregulatory interactions between a lymphoid and a nonlymphoid cell (NES = 3.124, size = 91), infectious disease (NES = 3.566, size = 88), hemostasis (NES = 3.447, size = 92), initial triggering of complement (NES = 4.136, size = 67), and leishmania infection (NES = 3.811, size = 78) (Fig. 7).

### Correlation analysis between HAMP expression and infiltrating immune cells

**Fig. 8:**
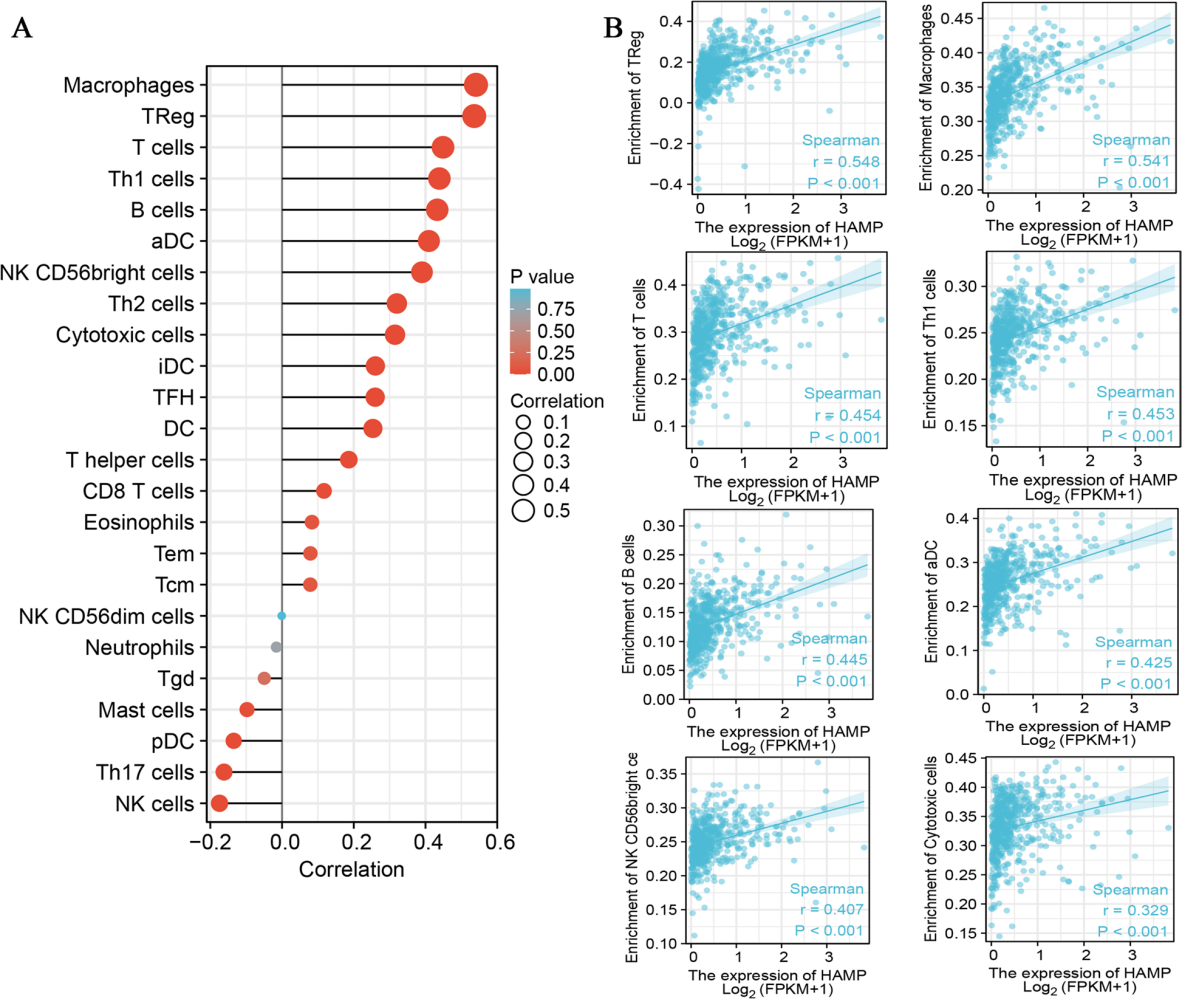
Correlation of hepcidin expression with immune infiltration level. **A** Correlation analysis between HAMP expression and the relative abundance of 24 immune cells. The size of the dots represents the absolute Spearman’s correlation coefficient values. **B** HAMP was significantly positively correlated with the infiltration of different immune cells in the TCGA database

We analyzed the correlation between HAMP expression and twenty-four types of infiltrating immune cells by ssGSEA. The results showed that HAMP expression was significantly positively correlated with TReg, macrophages, T cells, Th1 cells, B cells, aDCs, NK CD56bright cells, cytotoxic cells, Th2 cells, TFH cells, iDCs, DCs, T helper cells, and CD8 T cells. In contrast, HAMP expression was significantly negatively correlated with Th17 cells, NK cells, pDCs, and mast cells (Fig. 8A, 8B). In order to further explore the role of HAMP in various immune cell infiltration in ccRCC, we used the GEPIA and TIMER online website to carry out the relationships between HAMP and several immune marker sets, such as B cell, CD8^+^ T cells, T cells, Macrophages, M1/M2 macrophages, Tumor-associated macrophage (TAM), Monocytes, Neutrophils, NK, and DCs in ccRCC (Table 2). The results showed that the levels of most immune sets marking different T cells, B cells, TAMs, M2 macrophages, Monocytes, NK and DCs were associated with the HAMP expression in ccRCC.

**Table 2:** Correlation analysis between HAMP and markers of immune cells in TIMER and GEPIA

Cell type	Gene marker	None		Purity		Tumor		Normal	
		Cor	*P*	Cor	*P*	R	*P*	R	*P*
B cell	CD19	0.376	****	0.337	****	0.031	0.48	0.22	0.06
	CD38	0.425	****	0.411	****	0.37	****	0.32	**
CD8^+^ T cell	CD8A	0.382	****	0.353	****	0.22	****	0.48	****
	CD8B	0.406	***	0.337	****	0.17	****	0.74	****
Tfh	ICOS	0.383	****	0.359	****	0.24	****	0.49	****
	CXCR5	0.437	****	0.408	****	0.085	0.052	0.26	*
Th1	STAT4	0.220	****	0.185	****	0.091	*	0.48	****
	STAT1	0.307	****	0.277	****	0.35	****	0.15	0.2
	IFN-γ (IFNG)	0.391	****	0.365	****	0.35	****	0.29	*
	TNF-α (TNF)	0.249	****	0.219	****	0.1	*	0.25	*
Th2	CCR3	0.335	****	0.315	****	0.15	***	0.36	**
	STAT5A	0.395	****	0.373	****	0.23	****	0.46	****
Th9	IRF4	0.426	****	0.393	****	0.24	****	0.63	****
	SPI1	0.655	****	0.639	****	0.47	****	0.59	****
Th17	IL-21R	0.418	****	0.362	****	0.47	****	0.6	****
	IL-23R	0.218	****	0.171	***	0.12	**	0.59	****
Th22	CCR10	-0.045	0.302	-0.113	0.015	-0.073	0.097	0.17	0.15
	AHR	-0.0828	0.058	-0.113	0.501	0.04	0.36	0.37	**
Treg	FOXP3	0.501	****	0.482	****	0.29	****	0.41	***
	CD25(IL2RA)	0.404	****	0.378	****	0.51	****	0.42	***
	CCR8	0.385	****	0.359	****	0.21	****	0.46	****
T cell exhaustion	PD-1	-0.0138	0.761	-0.039	0.403	0.084	0.055	0.12	0.3
	PDCD1	0.430	****	0.409	****	0.2	****	0.42	****
	CTLA4	0.381	****	0.356	****	0.22	****	0.36	**
	LAG3	0.439	****	0.415	****	0.3	****	0.24	*
	TIM-3(HAVCR2)	0.139	0.001	0.115	0.014	-0.0056	0.9	0.22	0.058
	PDCD1LG2	0.269	****	0.228	****	0.21	****	0.25	*
	SIGLEC15	0.302	****	0.307	****	0.026	0.55	0.099	0.41
	TIGIT	0.418	****	0.398	****	0.28	****	0.39	***
Macrophage	CD68	0.456	****	0.477	****	0.3	****	0.6	****
	CD11b(ITGAM)	0.434	****	0.426	****	0.12	**	0.6	****
M1	INOS(NOS2)	-0.273	****	-0.332	****	-0.11	*	0.12	0.32
	IRF5	0.274	****	0.273	****	0.19	****	-0.037	****
	COX2(PTGS2)	0.062	0.151	0.017	0.710	-0.021	0.64	0.082	0.49
M2	ARG1	-0.010	*	-0.081	0.081	-0.022	0.61	0.17	0.16
	MS4A4A	0.412	****	0.386	****	0.37	****	0.48	****
TAM	CD80	0.479	****	0.452	****	0.43	****	0.34	**
	CD86	0.546	****	0.537	****	0.45	****	0.49	****
	CCR5	0.456	****	0.440	****	0.33	****	0.56	****
Monocyte	CD14	0.598	****	0.573	****	0.61	****	0.52	****
	CD115(CSF1R)	0.443	****	0.419	****	0.45	****	0.53	****
Neutrophil	CD66b(CEACAM8)	-0.107	0.013	-0.103	0.026	-0.0014	0.98	0.023	0.85
	CD15(FUT4)	0.033	0.447	-0.016	0.727	0.17	***	0.32	**
Natural killer cell	XCL1	0.432	****	0.400	****	0.17	***	0.41	***
	CD7	0.413	****	0.381	****	0.029	0.5	0.65	****
Dendritic cell	CD11c(ITGAX)	0.461	****	0.462	****	0.17	****	0.52	****

Tfh: Follicular helper T cell, Th: T helper cell, Treg: Regulatory T cell, TAM: Tumor-associated macrophage. None, Correlation without adjustment. Purity, Correlation adjusted by purity. Cor, R value of Spearman’s correlation. *P < 0.05; **P < 0.01; ***P < 0.001; ****P < 0.0001

**Fig. 9:**
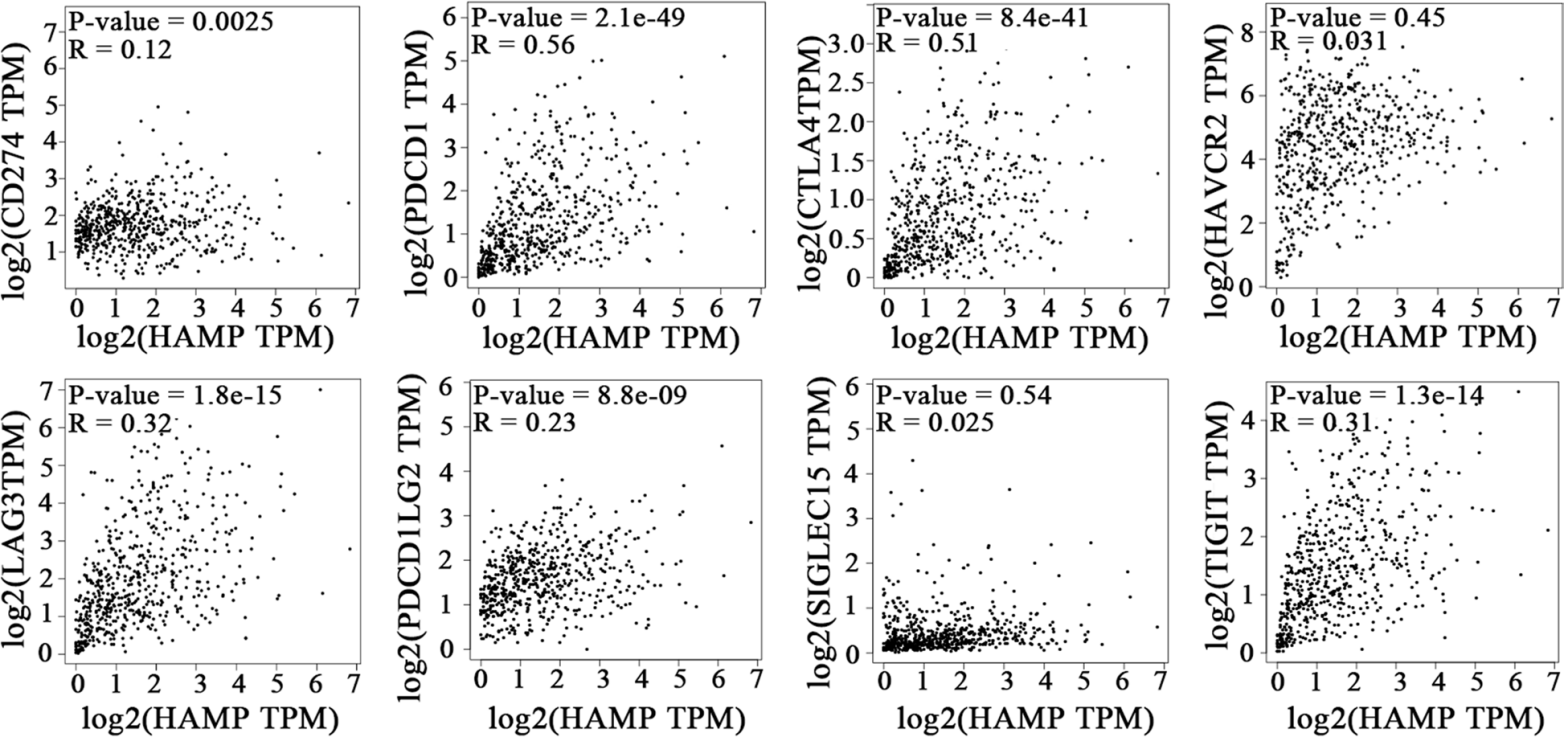
The correlation between HAMP expression and various immune markers, including CD274, PDCD1, CTLA4, LAG3, HAVCR2, PDCD1LG2, SIGLEC15, and TIGIT

We investigated the correlation between HAMP expression and various immune checkpoint markers in ccRCC, including PD-1, PD-L1, CTLA-4, LAG3, HAVCR2, PDCD1LG2, SIGLEC15, and TIGIT. The results showed that HAMP expression was significantly positively correlated with PDCD1, and CTLA-4 in ccRCC, with correlation coefficients of 0.56, and 0.51, respectively. The HAMP expression was not significantly correlated with CD274, LAG3, HAVCR2, PDCD1LG2, SIGLEC15, and TIGIT (Fig. 9).

## Discussion

In recent decades, along with the advancement of imaging technology, the detection rate of ccRCC has ascended steadily, placing a huge burden on health systems. At present, ccRCC remains a highly lethal malignancy and currently has few effective treatments. Surgical treatment is the gold standard in the treatment of localized renal tumors, but a proportion of patients still relapse. Although immunotherapy improved survival rates for advanced ccRCC, only a small percentage of patients are under a good response [5]. An effective prognostic biomarker is a key component in personalized and precision medicine and can prevent undertreatment or overtreatment of ccRCC patients.

An increasing number of studies have revealed that abnormal expression or activation of HAMP is a common phenomenon in multiple malignancies, and it has been demonstrated that HAMP expression is significantly associated with cancers, including [24–34] (Table 5). A recent study demonstrated that HAMP, as a tumor suppressor gene of hepatocellular carcinoma, downregulation contributes to proliferation, aggressiveness, and metastasis of hepatocellular carcinoma via the cyclin 4-dependent kinase-1/STAT3 pathway [35]. Sornjai et al. found that HAMP is upregulated and mediates human colorectal cancer cell growth [36]. Increased HAMP expression in non-small-cell lung cancer tissue and serum is associated with lymph node metastasis and tumor clinical stage [37]. Schwartz et al. demonstrated that HAMP in the tumor epithelium establishes an axis to sequester iron to maintain the nucleotide pool and sustain proliferation in colorectal tumors [38]. Wang et al. reported that HAMP is highly expressed in prostate cancer cells and can regulate cell proliferation, migration, and apoptosis by increasing intracellular iron transportation [39]. Meanwhile, Tesfay et al. found that prostate epithelial cells also synthesize HAMP and that the synthesis and secretion of HAMP are obviously increased in prostate cancer cells and tissue. The dysregulation of HAMP is correlated with prostate cancer growth and progression [40]. Blanchette-Farra et al. revealed that HAMP plays a vital role in regulating the growth of BRCA, and the combined expression of HAMP and its membrane target, ferroportin, predicts the prognosis of breast cancer [41]. Moreover, Orlandi et al. also found that the level of HAMP in plasma can be a noninvasive tool for predicting the prognosis of breast cancer [42]. However, the prognostic value of HAMP in ccRCC remains unclear.

In the present study, we performed systematic bioinformatics approaches to investigate the roles of HAMP and its association with immune cell infiltration in ccRCC. We revealed that HAMP was significantly upregulated in ccRCC tissues compared to normal samples, and overexpression of HAMP was correlated with poor clinicopathologic factors in ccRCC, suggesting that HAMP functioned as an oncogene in ccRCC. In addition, HAMP upregulation was associated with the prognosis of ccRCC. In view of the important role of HAMP in cancer, it may serve as a potential biomarker for ccRCC. Then, we executed GO and KEGG enrichment analyses to reveal that several immune-related pathways were significantly enriched, including “humoral immune response”, “immunoglobulin complex”, “immunoglobulin receptor binding”, and “cytokine–cytokine receptor interaction”. Interestingly, GSEA also found that the most enriched pathways were correlated with immunity, such as “immune system imbalance” and “adaptive immune system”.

Tumor-infiltrating immune cells are an indispensable part of the tumor immune microenvironment, and their composition and distribution are closely related to tumor prognosis [43, 44]. HAMP upregulation might be accompanied by increased immune surveillance and even less responsive to immunotherapy in the ccRCC microenvironment. We sought to investigate the relationship between the levels of immune cell infiltration and HAMP expression in ccRCC patients. The results showed that HAMP expression was significantly positively correlated with Tregs, macrophages, T cells, Th1 cells, B cells, aDCs, NK CD56bright cells, cytotoxic cells, and Th2 cells. These findings indicated that HAMP expression is closely associated with immune infiltration and plays a vital role in immune escape in the ccRCC microenvironment. Treg cells were initially defined as CD4 + T cells with high expression of CD25 [45]. Previous studies have shown that Treg cells abundantly infiltrate tumor tissues, which enhances antitumor immune responses and is often associated with poor prognosis in various types of cancer patients [46, 47]. Macrophages are key regulators of homeostatic tissue and tumor microenvironments and play important roles in clearing pathogens and maintaining tissue homeostasis [48]. Tumor-associated macrophages are abundant in many cancers, predominantly displaying an M2-like immunosuppressive function and promoting tumor progression and malignant metastasis [49]. T cell metabolism has a critical role in immune responses and may have a key role in antitumor immunity [50]. Arnold et al. found that the GM-CSF-IRF5 signaling axis in eosinophils promotes antitumor immunity through activation of Th1 cell responses [51]. B cells, as an integral component of the tumor microenvironment, exist in all stages of various cancers and play important roles in shaping tumor development [52]. Therefore, we inferred that HAMP might affect the prognosis of ccRCC patients by modulating immune infiltration.

Although our study can provide novel insights into the correlation between HAMP and ccRCC, inevitably, there are several limitations in this study. First, all data used in the present study were derived from public databases and were retrospective. Although the results indicate that HAMP expression could act as an independent prognostic factor in ccRCC patients, further experimental studies are required for validation.

## Conclusion

In this study, we performed comprehensive analyses of the expression and potential values of HAMP in ccRCC. Our findings demonstrated that HAMP may have potential as a biomarker in predict prognosis and the clinical treatment outcome of ccRCC patients. The high expression of HAMP was associated with worse clinical prognosis and more immune cell infiltration in ccRCC patients. So HAMP hold the expectation as a novel marker for identifying potentially eligible ccRCC patients for combinating with immunotherapy.

## Supplementary Information

**Additional file 1.**

**Additional file 2.**

**Additional file 3.**

**Additional file 4.**

**Additional file 5.**

**Additional file 6.**

**Additional file 7.**

**Additional file 8.**

**Additional file 9.**

**Additional file 10.**

**Additional file 11.**

**Additional file 12.**
