# Supplementary material for: High Hepcidin expression predicts poor prognosis in patients with clear cell renal cell carcinoma
Source: Diagn Pathol. 2022 Dec 31;17:100. doi: 10.1186/s13000-022-01274-9 (PMC9805116; doi:10.1186/s13000-022-01274-9)
Supplement: Supplementary file 1 — Additional file 1. [file 13000_2022_1274_MOESM1_ESM.docx]

**Table 5. The expression and mechanism of HAMP were studied in various tumors.**

| **Change of hepcidin** | **Pathogenetic mechanism** | **Type of tumor** | **Author** |
| --- | --- | --- | --- |
| Upregulated | Regulates iron transport by binding to and degrading ferroportin in macrophages, hepatocytes and enterocytes | Breast cancer | Pinnix, et al.^[26]^ |
| Downregulated | Activation of CDK1/STAT3 pathway； BMP/SMAD signaling | Hepatocellular carcinoma | Michael C Kew.^[27]^ |
| Upregulated | Regulating iron metabolism genes | Pancreatic ductal adenocarcinoma | Serrano, et al.^[28]^ |
| Upregulated | Down-regulation of ferroportin | Breast cancer | [Mark D Fleming](https://pubmed.ncbi.nlm.nih.gov/?size=200&term=Fleming+MD&cauthor_id=19074074).^[29]^ |
| Upregulated | Iron-restricted erythropoiesis | The early stages of prostate cancer | Ganz, et al.^[30]^ |
| Upregulated | Acts by promoting endocytosis and degradation of ferroportin, leading to the retention of iron in iron-exporting cells and decreased flow of iron into plasma | [Colorectal Cancer Patients.](https://pubmed.ncbi.nlm.nih.gov/34249766/) | Shao, et al.^[31]^ |
| Upregulated | Sunitinib-related resistance | Renal cell carcinoma | Peng, et al.^[32]^ |
| Upregulated | Ferroptosis-related | Skin cutaneous melanoma | Ping, et al.^[33]^ |
| Upregulated | BMP/Smad4/Hamp pathway | Non-small cell lung cancer | Yang, et al.^[34]^ |
| Upregulated | Acts by promoting endocytosis and degradation of ferroportin | Clear cell renal cell carcinoma | Our study. |
