# Supplementary figures and images for: High Hepcidin expression predicts poor prognosis in patients with clear cell renal cell carcinoma
Source: Diagn Pathol. 2022 Dec 31;17:100. doi: 10.1186/s13000-022-01274-9 (PMC9805116; doi:10.1186/s13000-022-01274-9)

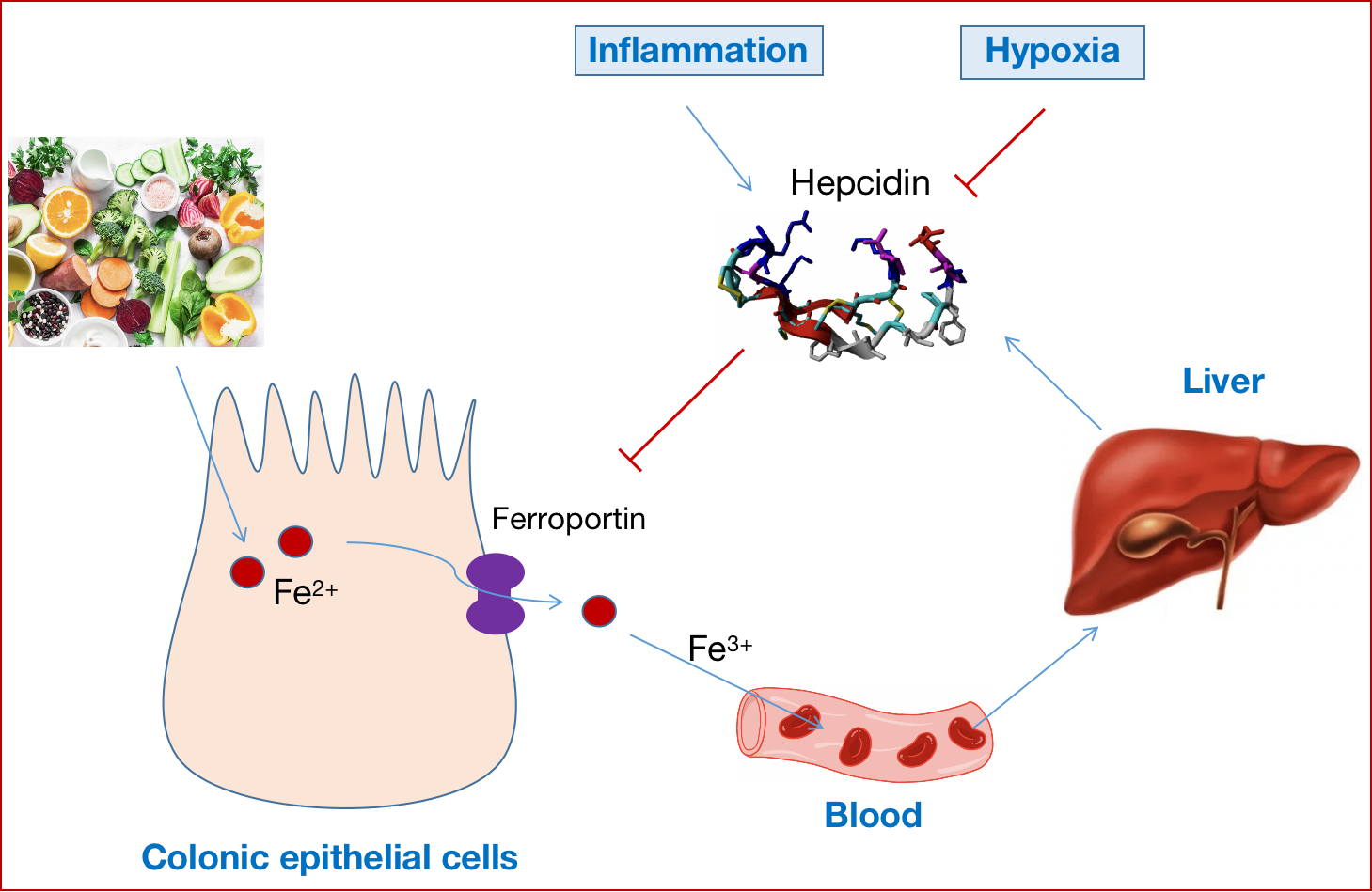

Supplement: Supplementary file 3 — Additional file 3. [file 13000_2022_1274_MOESM3_ESM.tif]

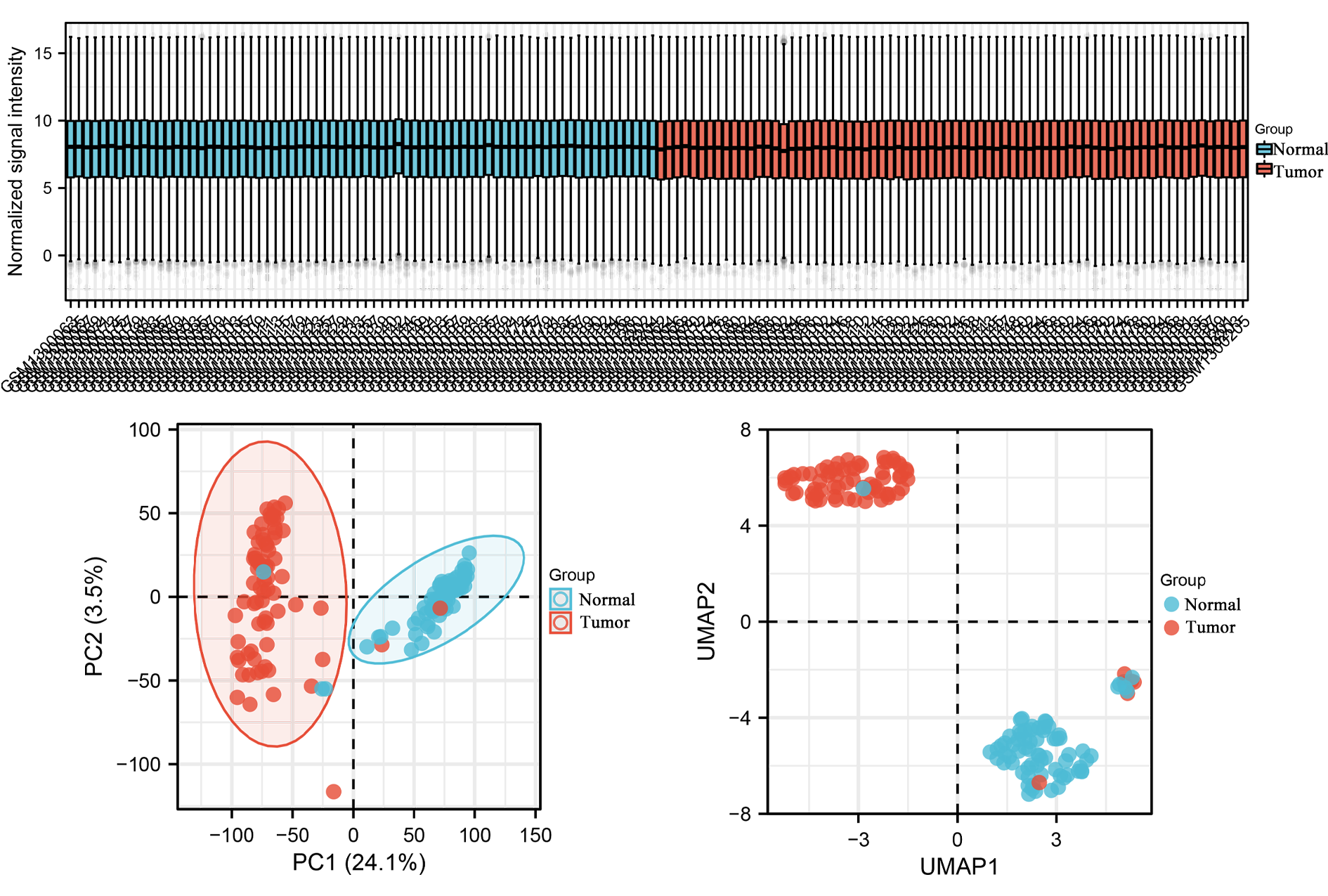

Supplement: Supplementary file 4 — Additional file 4. [file 13000_2022_1274_MOESM4_ESM.tif]

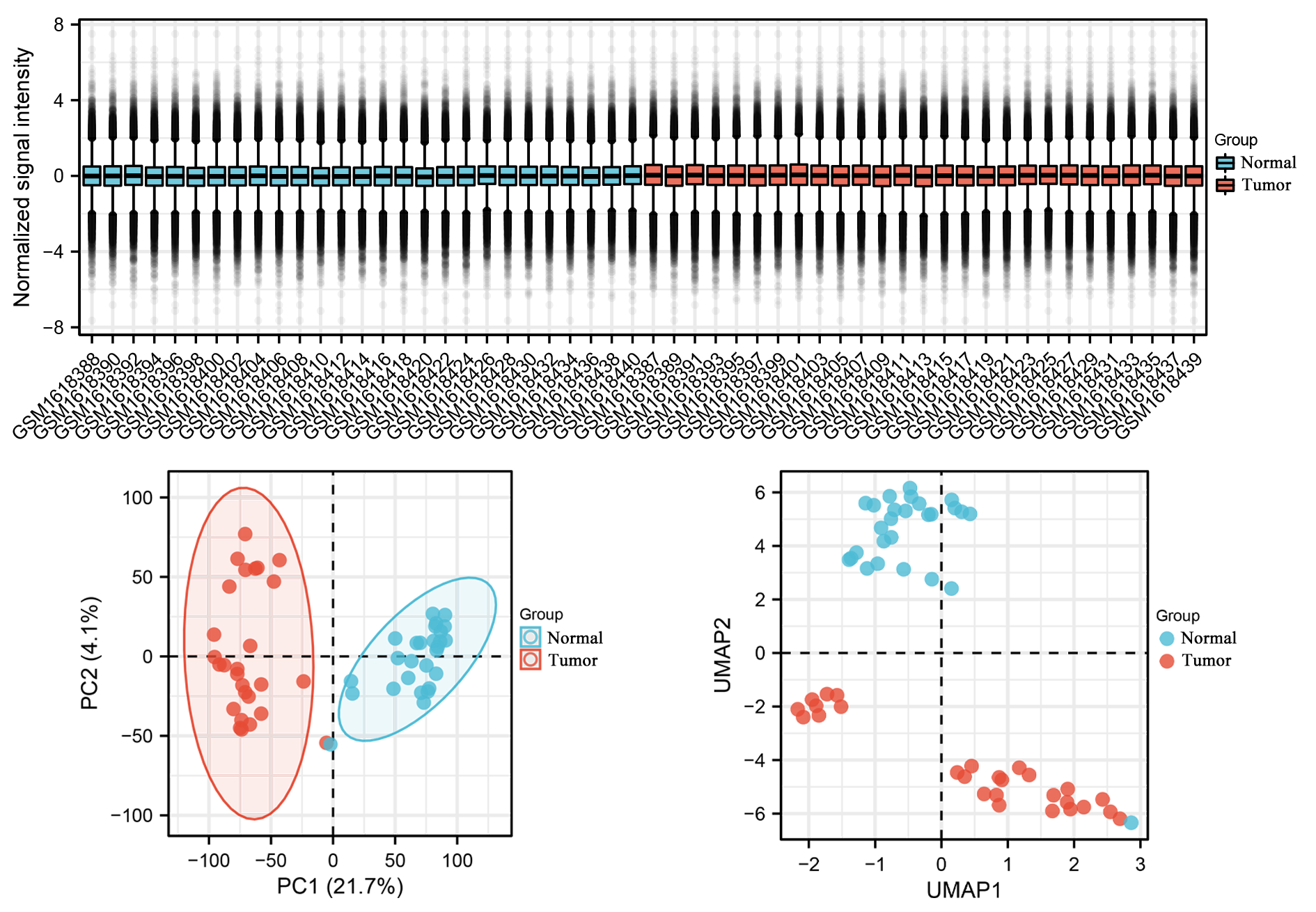

Supplement: Supplementary file 5 — Additional file 5. [file 13000_2022_1274_MOESM5_ESM.tif]

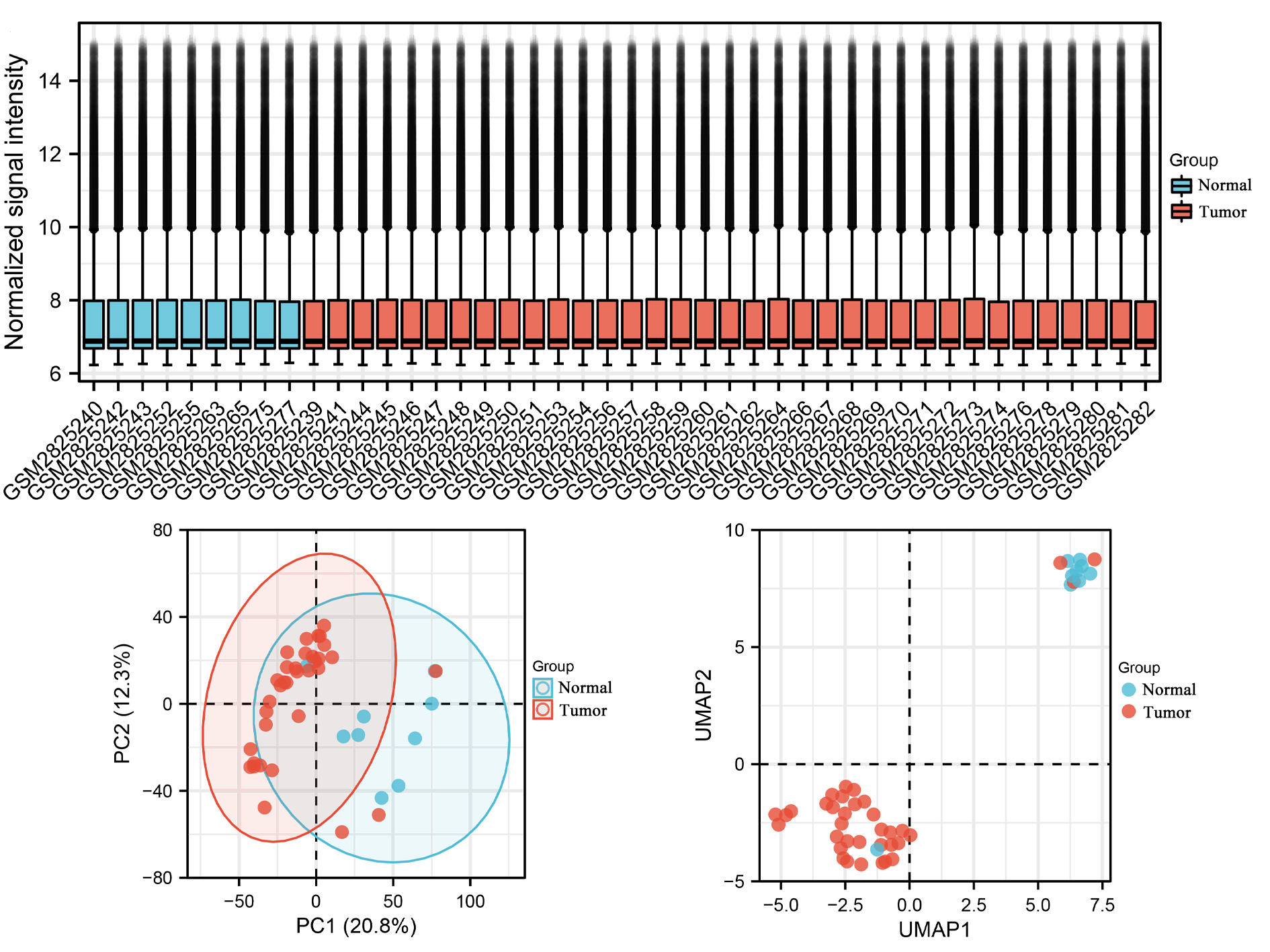

Supplement: Supplementary file 6 — Additional file 6. [file 13000_2022_1274_MOESM6_ESM.tif]

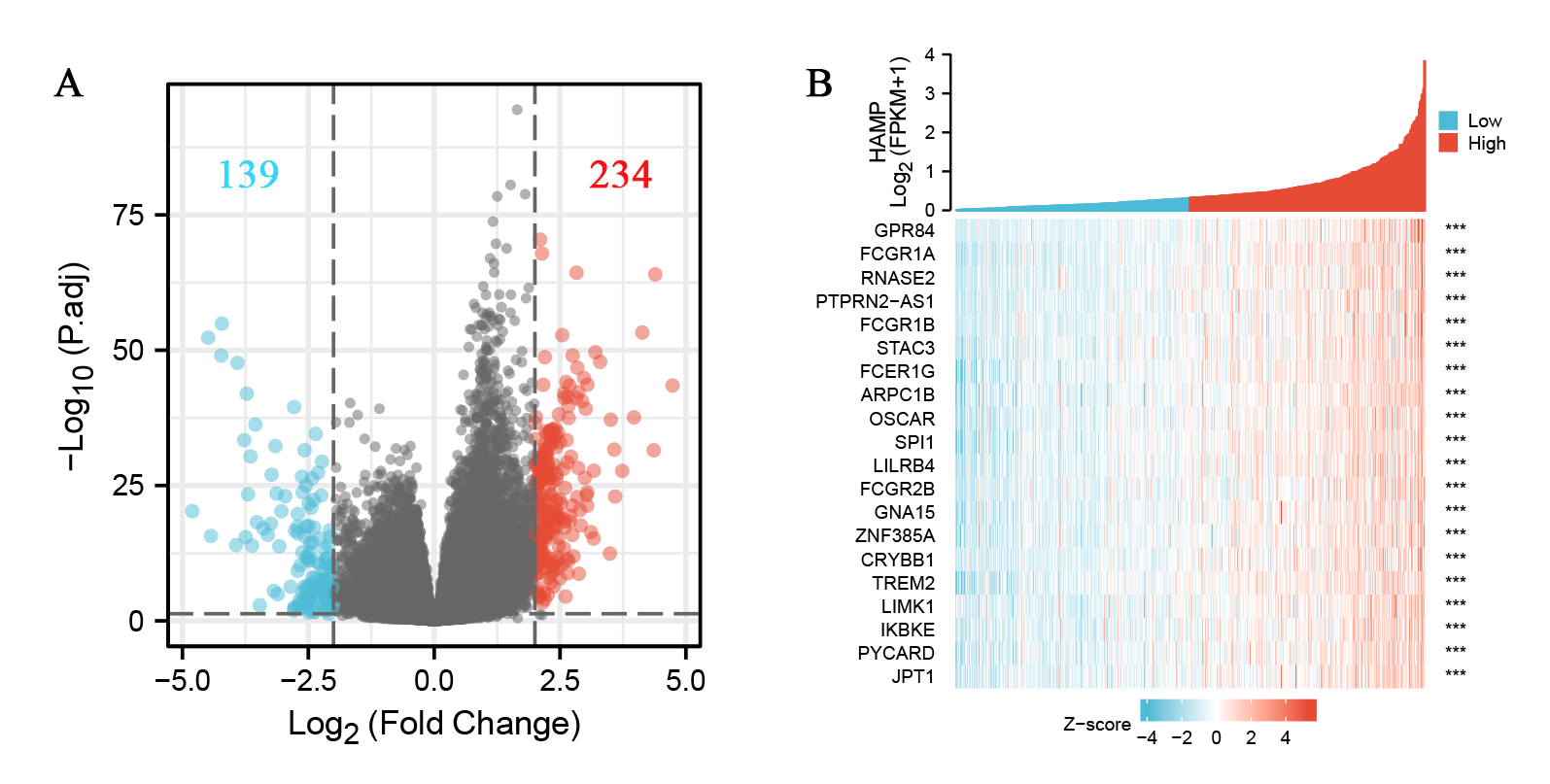

Supplement: Supplementary file 7 — Additional file 7. [file 13000_2022_1274_MOESM7_ESM.tif]

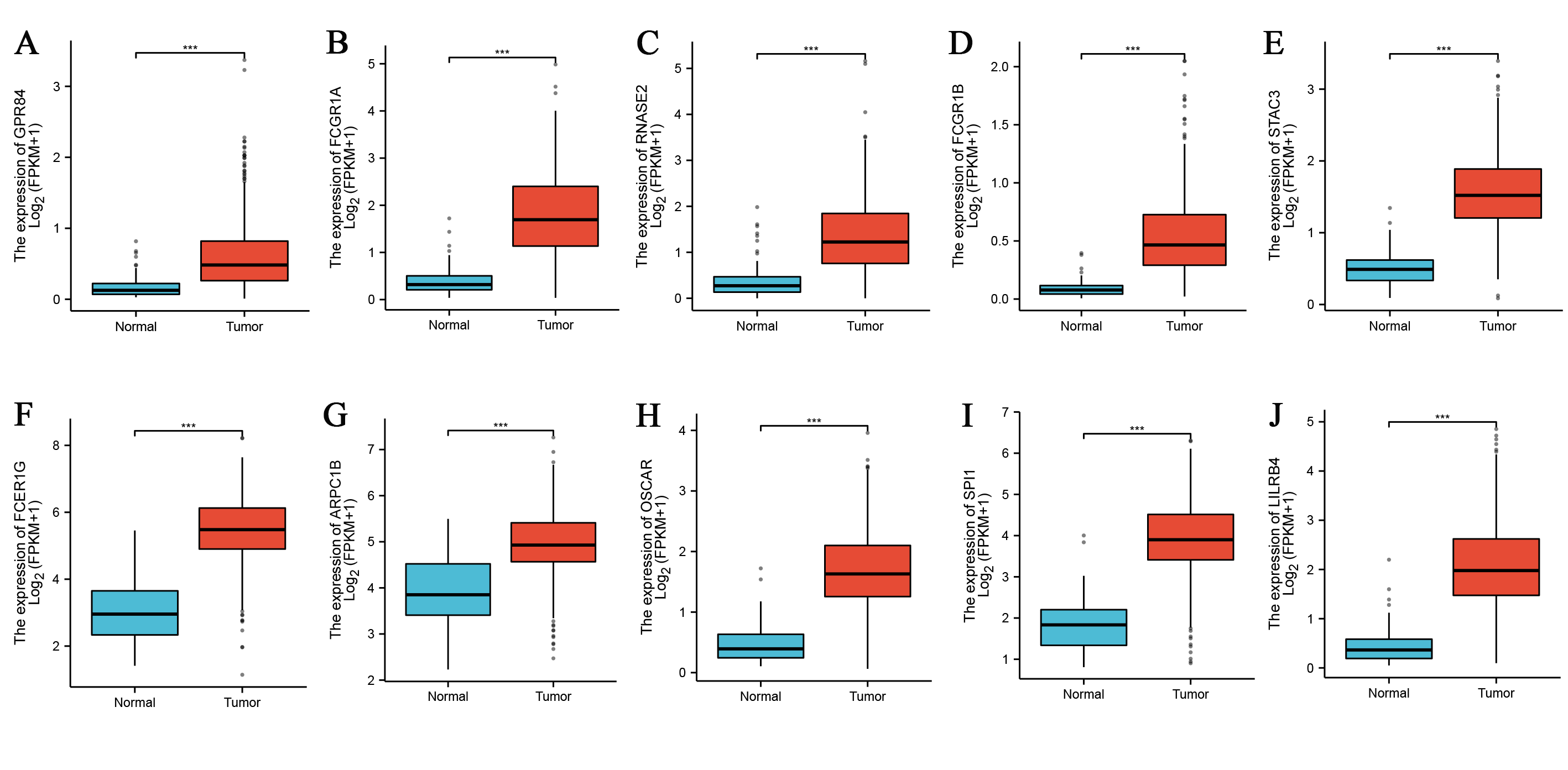

Supplement: Supplementary file 9 — Additional file 9. [file 13000_2022_1274_MOESM9_ESM.tif]

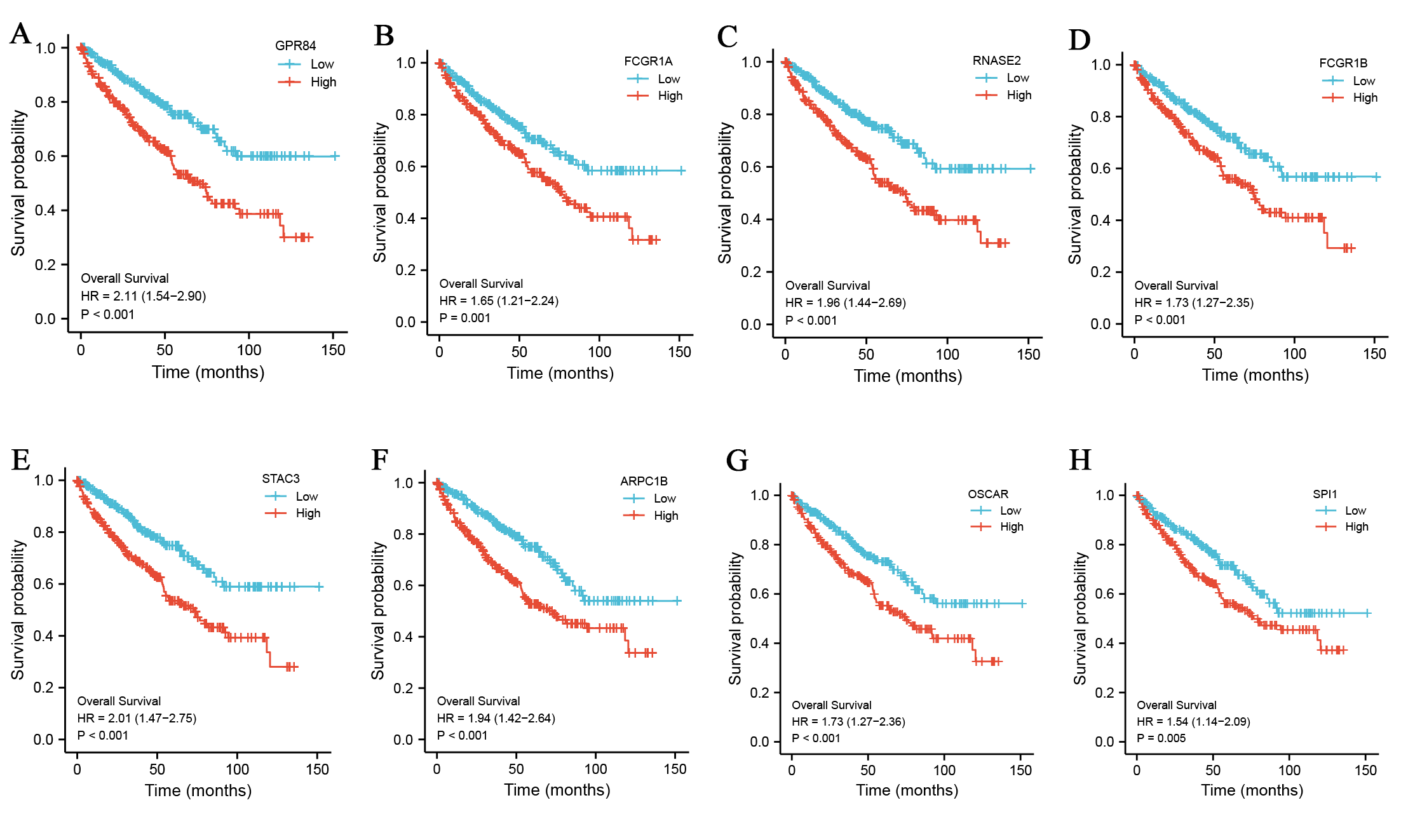

Supplement: Supplementary file 10 — Additional file 10. [file 13000_2022_1274_MOESM10_ESM.tif]
